# Design of the Global Health chemical diversity library v2 for screening against infectious diseases

**DOI:** 10.1371/journal.pntd.0011799

**Published:** 2023-12-27

**Authors:** Caroline Wilson, J. Mark F. Gardner, David W. Gray, Beatriz Baragana, Paul G. Wyatt, Alex Cookson, Stephen Thompson, Cesar Mendoza-Martinez, Michael J. Bodkin, Ian H. Gilbert, Gary J. Tarver

**Affiliations:** 1 Drug Discovery Unit, Wellcome Centre for Anti-Infectives Research, Division of Biological Chemistry and Drug Discovery, University of Dundee, Dundee, United Kingdom; 2 AMG Consultants Ltd, Discovery Park House, Ramsgate Road, Sandwich, Kent, United Kingdom; KU Leuven, BELGIUM

## Abstract

There is a need for novel chemical matter for phenotypic and target-based screens to find starting points for drug discovery programmes in neglected infectious diseases and non-hormonal contraceptives that disproportionately affect Low- and Middle-Income Countries (LMICs). In some disease areas multiple screens of corporate and other libraries have been carried out, giving rise to some valuable starting points and leading to preclinical candidates. Whilst in other disease areas, little screening has been carried out. Much screening against pathogens has been conducted phenotypically as there are few robustly validated protein targets. However, many of the active compound series identified share the same molecular targets. To address the need for new chemical material, in this article we describe the design of a new library, designed for screening in drug discovery programmes for neglected infectious diseases. The compounds have been selected from the Enamine REAL (REadily AccessibLe) library, a virtual library which contains approximately 4.5 billion molecules. The molecules theoretically can be synthesized quickly using commercially available intermediates and building blocks. The vast majority of these have not been prepared before, so this is a source of novel compounds. In this paper we describe the design of a diverse library of 30,000 compounds from this collection (graphical abstract). The new library will be made available to laboratories working in neglected infectious diseases, subject to a review process. The project has been supported by the Bill & Melinda Gates Foundation and the Wellcome Trust (Wellcome).

## Author summary

Screening of diverse compound libraries is a powerful way to find chemical start points for drug discovery programmes. There is a lack of such chemical libraries available for neglected infectious diseases and other neglected areas such as non-hormonal contraception. In this paper we describe the design of such a library of 30,000 compounds (graphical abstract). The aim was to develop a library of diverse chemical matter unlikely to have been screened in these disease areas. The compounds were selected from the REAL (REadily AccessibLe) Enamine library, a virtual library of 4.5 billion compounds. The methodology for selection of the compounds is described, along with a detailed analysis of the physicochemical properties of the compounds and comparison to other libraries that we have assembled. This shows that the library contains hit/lead-like compounds suitable as chemical start points, and whilst it covers a similar space to some other libraries, the material within the library is mainly different to other libraries. Copies of the library will be made available for screening; the process for gaining access to this library is described.

## Introduction

Neglected infectious diseases have a massive impact, particularly in Low- and Middle-Income Countries (LMICs). For the vast majority of these diseases, there is an urgent need for new medicines, due to lack of treatments, current medicines having inadequate efficacy or to emerging resistance [1]. To facilitate drug discovery programmes, there is a need for suitable compound libraries, for screening both phenotypically and against validated drug targets. For many of these disease areas, there are very few well validated protein targets, so much of the screening has been conducted phenotypically. Fortunately, recent successes in target identification of phenotypic hit compounds are identifying good drug targets in some of these diseases [2]. In some areas, such as malaria, tuberculosis and the kinetoplastid diseases, significant numbers of compounds have been screened and hits followed up using corporate libraries and those available through academic groups and product development partnerships [3–6]. The need remains for new screening libraries occupying novel chemical space to find new phenotypic hits and new chemical matter for precedented protein targets. Analysis of phenotypic actives in malaria, TB and other neglected diseases indicates that certain targets are inhibited by a variety of different chemotypes; some of these are good targets, but for which there are already compounds in development; some of the targets are low priority targets as there are already compounds in development; and some of these are not targets of interest. For example: in *Trypanosoma cruzi*, a very high proportion of new hits either inhibit CYP51 or cytochrome b; in *P*. *falciparum*, DHODH, PI4K and ATP4 are common targets; and in TB Qcrb and Mmpl3 are common targets.

In 2014 the Drug Discovery Unit (DDU) at the University of Dundee, with support from the Bill & Melinda Gates Foundation, constructed a 70,000 library of chemically novel, commercially available compounds for screening against priority pathogens (S1 Table). This library was specifically constructed to be within commonly accepted good physiochemical properties for drugs and was distributed to a number of organisations for screening. It was profiled in 24 assays across numerous pathogens, including malaria, TB, Chagas, HAT, Schistosomiasis, Wolbachia, Toxoplasma, and a number of target-based screens. Subsequently, the program was funded to build a second ~14,000 library consisting of small molecular weight, polar compounds as this chemical space was under-represented in traditional screening libraries. The small polar library has been assayed in 19 screens. Although analyses of the impact of these two libraries is still ongoing, nearly all the screens delivered high-quality drug leads that are now being further developed. Several publications, poster and grant applications demonstrate the library has delivered options for initiating drug discovery projects [7–15].

Given this success there is interest in building additional libraries for screening with our partners. We have designed a high-quality chemical library with the key feature that the chemical space is different from both previous libraries. The source of new chemical diversity for construction of this new library was Enamine as they offer access to billions of “virtual” compounds [16] where there is a high chance that they can be prepared using known chemistry and building blocks/ intermediates that are commercially available.

The DDU and AMG Consultants generated a second version of the Global Health Chemical Diversity Library (GHCDL_V2) of 30,000 compounds, with synthesis being carried out by Enamine from their REAL library. This work has been supported by the Bill & Melinda Gates Foundation and Wellcome. It will be screened in phenotypic and target-based assays against priority pathogens found in LMICs and also for non-hormonal human contraception (another area of unmet clinical need), with the aim of delivering hits for drug discovery projects and/or identifying new drug targets.

The library was selected to be chemically diverse while retaining physicochemical properties appropriate for hit or lead discovery across a wide range of diseases. To maximize the novelty of the library and to limit the chance compounds have already been screened against priority pathogens, we focused on bespoke synthesis of novel compounds selected from virtual compounds in the Enamine REAL (REadily AccessibLe) library which consisted of 4.5Bn compounds which comply with the Ro5 [17] and Veber [18] criteria. The compounds were made on a non-exclusive basis to minimize cost and to facilitate follow up of hits. Enamine offers a hit expansion service, where analogues of the hits in Enamine’s Screening Collection can be purchased and analogues from the REAL Database and REAL Space can be synthesised on demand. Follow-up libraries can also be designed using monomers in Enamine’s collection, depending on the outcome from any particular screen. Whilst many drugs are natural products or based on natural products, we decided to focus on small molecules which could be rapidly followed up by chemical synthesis or purchase of analogues.

## Methods: Design of the library

### Selection of reactions from a basis set and filtering for alerts

The first step in the library design was to identify reactions and building blocks that were of interest contained within Enamine’s REAL library. To select the reactions a small sub-set of Enamine’s REAL library (basis set) was built. The basis set was built using Enamine’s building blocks and the chemistry that Enamine can use. The building blocks were then enumerated with small reagents. For example, an amide bond formation reaction where 100 amines and 200 acids are available would be represented by 299 ‘basis product’ amides consisting of the 100 amines enumerated with a low molecular weight acid and the 199 additional acids enumerated with a low molecular weight amine. This gave a basis set from Enamine’s REAL library of 4.5Bn compounds grouped into 271 compound libraries and divided by chemistry and building block types. The compounds were flagged with structural alerts from published pan-assay interference compounds (PAINS) and the in-house Drug Discovery Unit structural alerts set supported by additional structural alerts from Lilly [19]. The PAINS are compounds that are often false positives in assays. The structural alerts are to remove compounds with functional groups that are associated with toxicity (such as aromatic nitro groups) and those with chemically reactive functionality, such as acid chlorides. Full details of the PAINS and the reactive/ toxic functionality are in the supplementary data of reference [19]. Some additional filters were used for this library and are included in the supporting information (S3 Table). A Data Warrior [20] file was then created with eight examples for each reaction ID; this included three of the lowest molecular weight examples and five other random examples from each reaction selected using an algorithm. This data was then used to guide selection by eye to further identify which reactions to include and exclude based on the product chemotypes. Four people assessed the file and scored each compound library (represented by eight compounds) for retention or rejection. Differences of opinion were resolved by discussion, to retain a total of 165 of the 271 compound libraries. Following this a small number of additional alerts were added (e.g. thioethers & sulfoxides) as suggested following internal discussion and consultation with a number of parties with potential interest in screening the library. The reactions selected from this basis set were then used to create the super-set described in the next section.

### Selection from super-set

A request was then sent to Enamine for an enumerated super-set of compounds from the reactions selected from the basis set meeting wide physicochemical properties (MWt < = 450, logP < = 5, HBD < = 4, HBA < = 8, Rotatable bond count < = 8) which lie within Ro5 [17] and Veber [18] criteria. Some compound libraries (i.e., reaction sets) are compatible with vast compound sets; where this was the case, we requested a maximum of 5 million compounds randomly selected from these sets. This library was designed to be an all-encompassing library of any compound that may possibly be wished for within the physicochemical property range.

In practice, 5 million per compound library was too high a number to be tractable so we carried out a crude filtering to a maximum of 250K compounds per compound library using random selection from all that passed the criteria MWt 320–420, logP 0–4.5, HBD 0–3, HBA 0–8, Rotatable bonds 1–8 and some additional structural filters (some examples, naphthalene, ester, bromine, sulfonimadamide, phthalimide), resulting in a set of 25.5 million compounds for further consideration. At this stage a diversity algorithm was applied to each compound library in turn. The diversity algorithm in RDKit as implemented in KNIME [21] was applied. This is based on a MaxMin algorithm [22]. The number of compounds to be selected from each compound library was chosen based on an assessment of the mean pairwise similarity within each compound library. This reduced the total compound set to 970K.

The properties of the library were then discussed with potential users of the library (DNDi, MMV, TB Alliance). As modest cost of goods is a major factor in successful medicines for LMIC infectious diseases, we have chosen to have 70% of the new library made up of simple chemistry with 1–2 steps and no expensive building blocks or requirement for specialist purification (s-REAL set). The remaining 30% are more complex needing either multistep synthesis procedures, or use of expensive building blocks or the products require special purification (m-REAL set). The agreed property ranges were as follows:- MWt 320–380, slogP 1–3, HBA 0–8, HBD 0–3, SFI [23] (clogD + #Arom) 2–6, Rotatable Bonds 1–7. The conclusion from the discussion with the potential users on charge was that charge tended to be added later in the hit/lead optimisation process to aid optimisation of pharmacodynamic properties, so the majority of the library is neutral: neutrals– 60%, bases– 33%, acids 7%.

To ensure novelty in the Malaria and Tuberculosis diseases a StarDrop model that MMV use to flag known antimalarial fragments was applied and compounds with > 0.5 Tanimoto similarity to the TBDA disclosed set of 12,000 compounds were removed. This disclosed set is a set of 12,000 compounds with some recorded activity against *Mycobacterium tuberculosis* in a whole cell assay maintained by TBDA. The similarity was calculated using the Morgan fingerprints obtained from the Morgan algorithm as available in the KNIME implementation of RDKit with radius of 2 and 1024 bits. The library was filtered using the agreed properties and diversity sampling was used to select a diverse set of ~100K compounds.

Compounds commercially available from other sources, for example MolPort, were removed to give a list of 55K compounds. A selection of the set was checked by eye by the core project team and DDU chemists and further alerts flagged (S3 Table) and applied to give a list of 52.5K compounds, the chemical diversity analysis of this set is discussed in the next section. The final step was to select a random 45K of the remainder ensuring a split of 70:30 simple/complex chemistry compounds.

**Fig 1:**
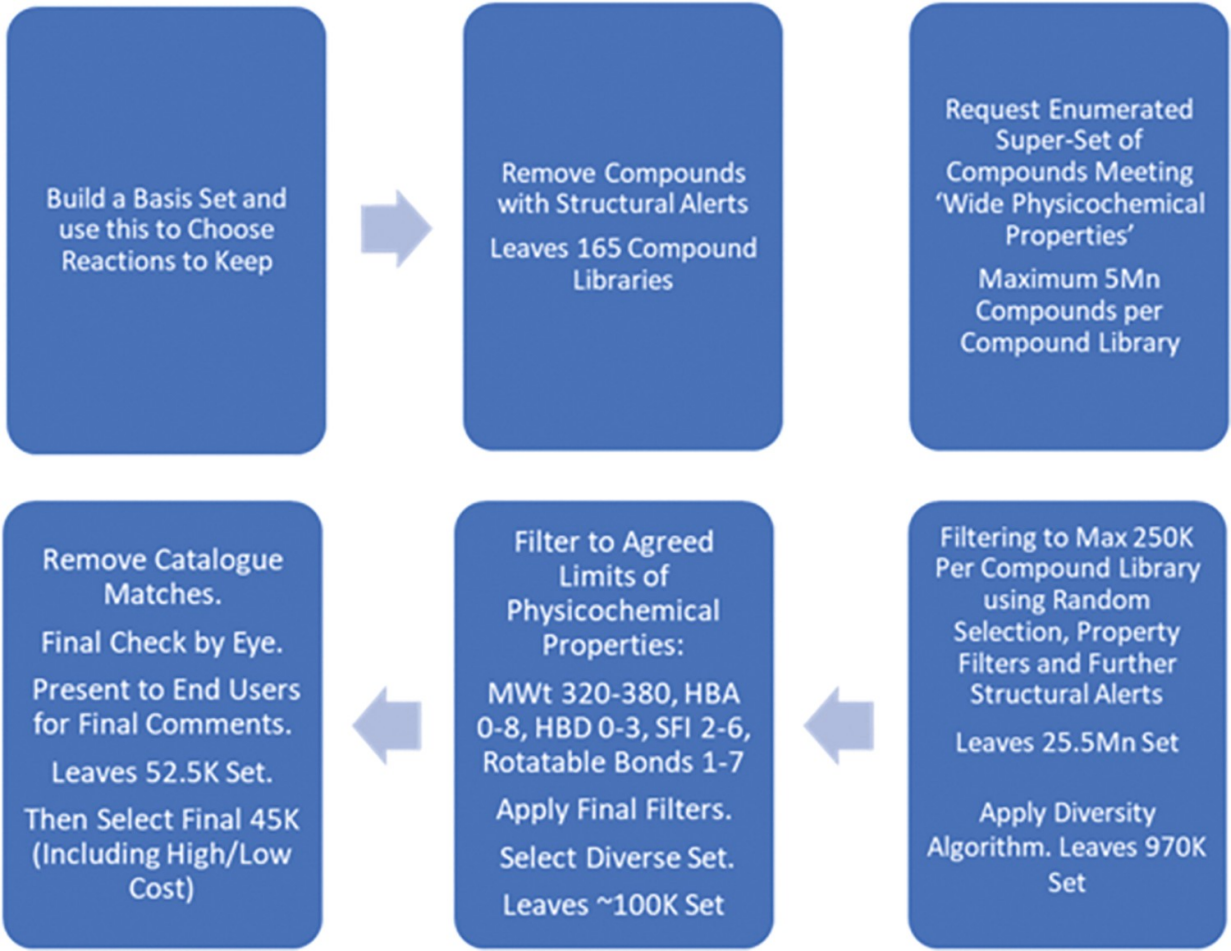
Compound selection process.

The overall selection process is shown graphically in Fig 1.

### Chemical diversity analysis

**Fig 2:**
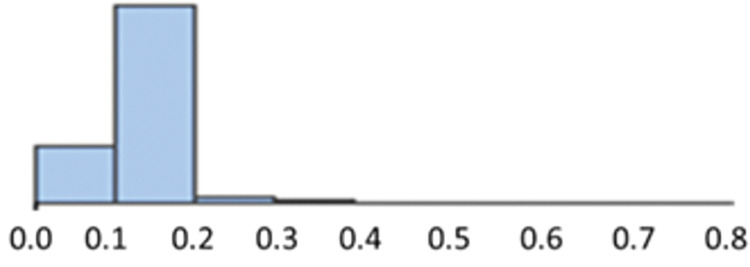
Similarity of pairs in the 52.5K compound set.

A diversity algorithm was applied to the 52.5K set and this indicated that this library has high level of diversity when compared to the Approved Drugs file of 1,578 drugs in Data Warrior. Diversity was analysed by calculating a fingerprint (Morgan fingerprint in RDkit) for each compound and calculating a full similarity matrix (using Tanimoto similarity) between all compounds (ignoring self-identity). The histogram was plotted and the mean similarity calculated for the full matrix (n x n-1 similarity values). A histogram of similarity between all pairs of compounds in the 52.5K set is shown in Fig 2.

### Comparison of s-REAL and m-REAL sets

It was possible to reduce cost of the library by focusing on simpler molecules only. The simpler molecules were compounds with simple chemistry with 1–2 steps and no expensive building blocks or requirement for specialist purification (s-REAL set). However, a comparison of the properties and range of scaffolds of the simple compounds and the more complex compounds showed that the complex set adds significant extra diversity (see Diversity Analysis below). The complex set were compounds needing either multistep synthesis procedures, or use of expensive building blocks or the products require special purification (m-REAL set). The more complex compounds have higher sp^3^ character and number of saturated rings, and lower number of rotatable bonds and phenyl rings. Moreover, they offer an increased number of additional Murcko [24] scaffolds. In the Murcko framework analysis comparing the addition of 6,000 complex compounds or 6,000 more simple compounds to a background of 24,000 simple compounds, the complex compounds add 50% more Murcko frameworks compared to the additional simple compounds.

**Fig 3:**
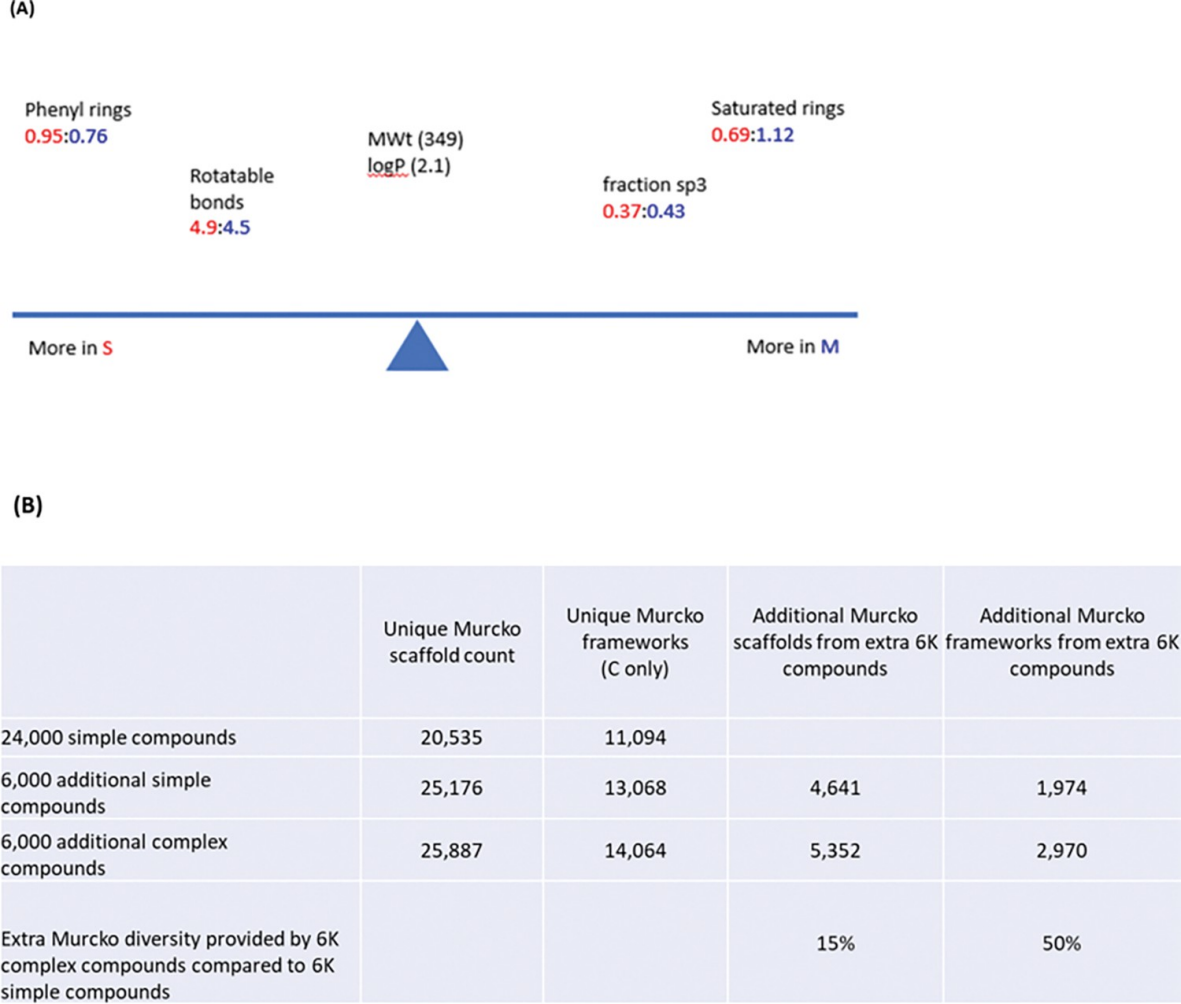
Property differences between Simple and Complex structures in a subset of the Enamine Real database and diversity analysis. (A) Property differences between simple (S) and complex (M), (B) Murcko framework analysis comparing the value of an additional 6,000 complex compounds to an additional 6,000 simple compounds on a base of 24,000 simple compounds. The complex compounds add 15% more Murcko scaffolds and 50% more Murcko frameworks compared to the simple compounds.

The extra diversity and moving into new areas of chemical space is important. There is a need to find new targets, which are relatively infrequently found using the current screened chemical matter. Therefore, we need to extend the chemical space in which we are screening, to identify and tackle new drug targets. The more complex set of compounds extends the chemical space in the library. In addition, the properties of the more complex set are in more developable chemical space [25], with higher sp^3^, lower number of rotatable bonds and lower number of phenyl rings (Fig 3). Therefore, our conclusion is that the more complex set does add significantly to the diversity of the GHCDLv2.

## Results

### Compound synthesis process and library construction

**Fig 4:**
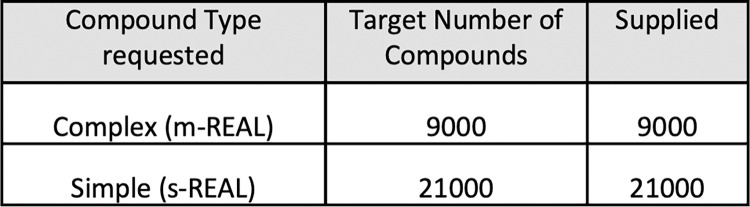
Final library make up of simple and complex structures.

A set of 30,000 compounds were selected for synthesis at Enamine and a 15,000 set was a reserved set to back-fill the library as it was anticipated that some chemistry would fail. Following agreement with funding partners the library was constructed by Enamine in Kyiv from late December 2022 until June 2023. As needed additional compounds were supplied to replace failed compounds in order to supply the full 30K library (S2 Table). There was an overall synthesis success rate of 81.7% with an 77.1% success for the complex (m-REAL) and 83.7% for the simple (s-REAL) compounds. The final composition of the library is shown in Fig 4. Compounds that were synthesised with metal catalyst were purified further with metal scavengers such as SiliaMetS DMT and SiliaMetS TAAcOH to remove metal contaminants. A set of 88 compounds containing mainly compounds that were synthesised using metal catalysts and then purified using metal scavengers were screened in an assay in the DDU which is very sensitive to metals and as a result has given false positive results. The results were that only 2 compounds from this set of 88 compounds had very weak potency so this indicates that this library could be screened in metal sensitive assays. The final materials were transferred to 2D barcoded latch rack vials and diluted in DMSO to 10 mM for shipping. Upon arrival 1% of the library was analysed for purity using LCMS. The solutions were plated to working plates to allow acoustic dispensing to generate screening plates for assays.

### Properties of final library (GHCDL-V2)

**Fig 5:**
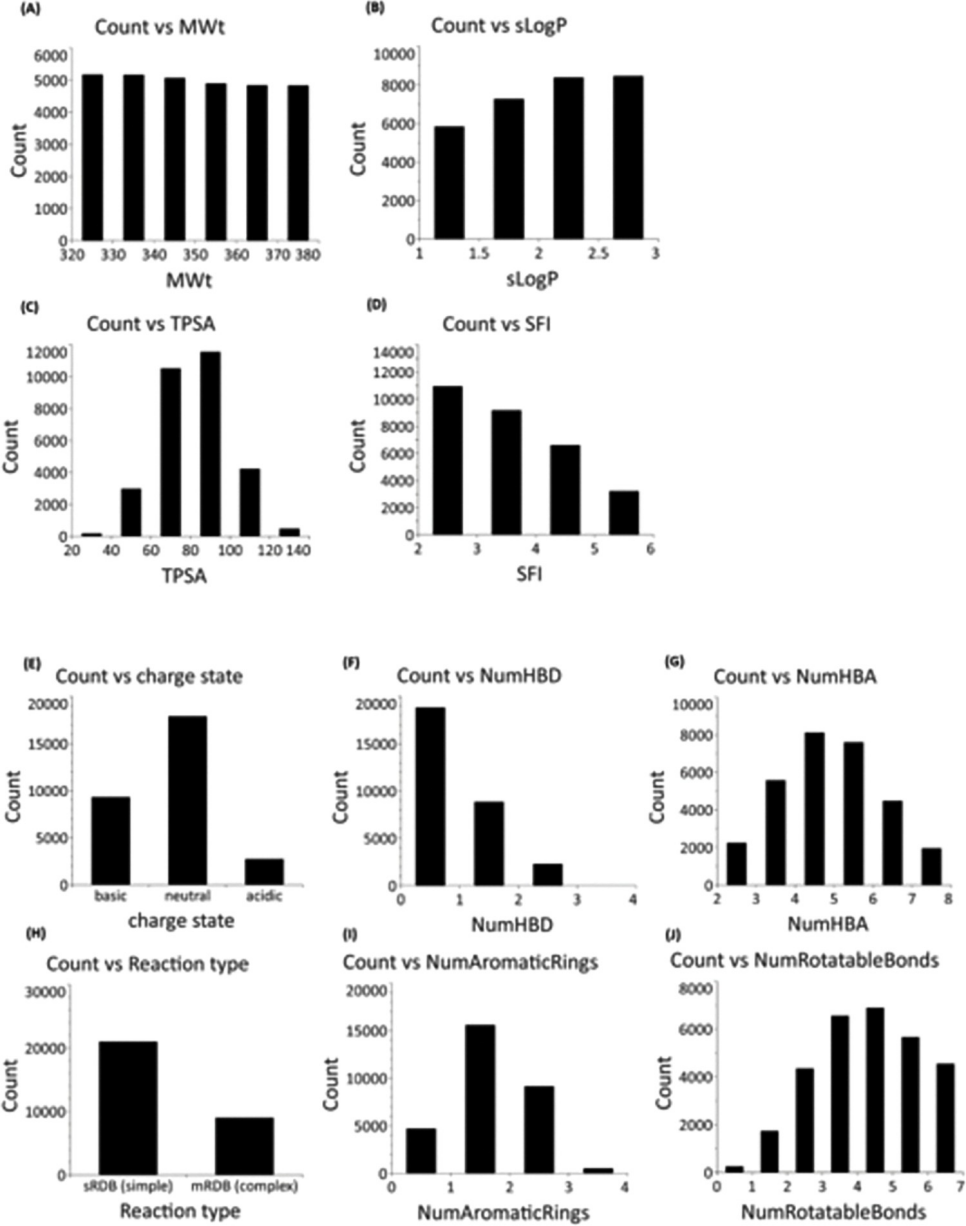
Properties of the 30K set. (A) Binned MWt 320–380, (B) binned sLogP 1–3, (C) binned TPSA 20–140, (D) binned SFI 2–6 (E) charge class, (F) number of HBD 0–3, (G) HBA 2–8, (H) synthetic type M (complex), S (simple), (I) number of aromatic rings 0–4, (J) number of Rotatable bonds 1–7.

The properties of the final 30K set were analysed by binned properties (Fig 5). The properties of the library were within lead-like property space, [26,27]. The molecular weight range was 320–380 with similar numbers of compounds in each bin, the sLogP was 1–3 and TPSA 20–140 with the maximum number of compounds in the 80–100 bin. The SFI (Solubility Forecast Index [17], clogD + number aromatic rings) ranged from 2–6 with decreasing numbers of compounds in each bin as SFI increased. The neutral compounds accounted for 60% of the set, bases 31% and acids 9%. HBA ranged from 2–8, HBD 0–3 and the number of rotatable bonds ranged from 1–7. The percentage of simple compounds was 70%, complex compounds 30%.

### Properties of GHCDL-V2 compared to the first GHCDL library and the small polar library

**Fig 6:**
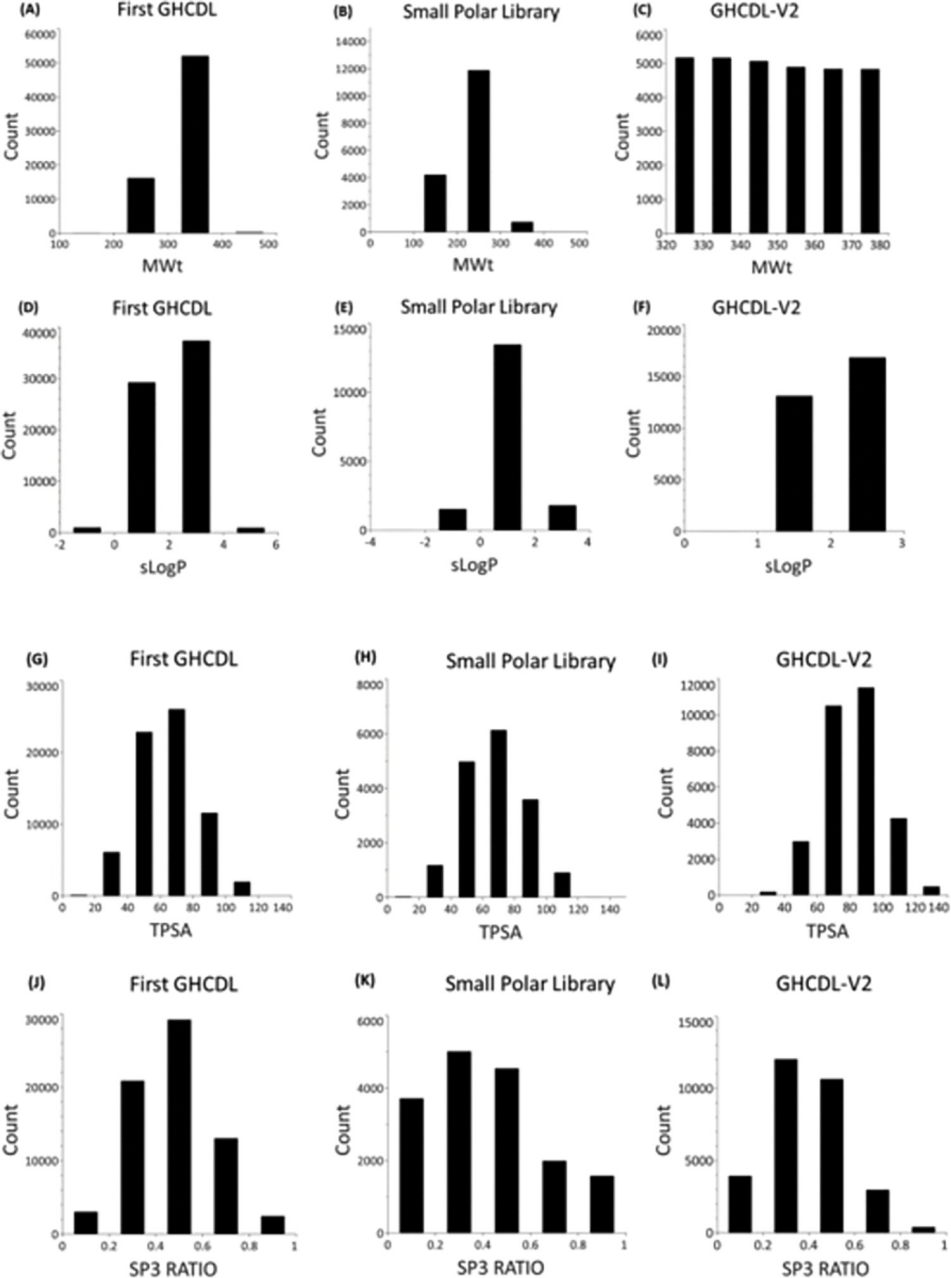
Properties of the first GHCDL, small polar library and GHCDL-V2 (A-C) Binned MWt, (D-F) binned sLogP, (G-I) binned TPSA (J-L) binned SP3 ratio.

Properties of the first GHCDL library and the small polar library were analysed by binned properties and compared to the 30K set (GHCDL-V2) (Fig 6). The first GHCDL library and the small polar library have a wider molecular weight and sLogP range than the GHCDL-V2 library, The range of TPSA of the first GHCDL library and the small polar library were the same (0–120), the TPSA for the GHCDL-V2 library was higher (20–140). The sp^3^ ratio of the first GHCDL library and the GHCDL-V2 were very similar, for the small polar library there was a larger proportion of compounds in the lower bins 0–0.2 and 0.2–0.4.

### Comparison of unique Murcko frames between the first version of GHCDL and GHCDL-V2

A calculation of the number of unique Murcko [24] frames done using RDKit as implemented in KNIME^21^ using the ‘RDKit Find Murcko Scaffolds’ with the ‘Create Frameworks’ toggle set to OFF. Compared to the first version of GHCDL analysis shows there are a higher percentage of unique Murcko frames in GHCDL-V2 88% vs 58%, when a random set of 30K was chosen from the first version of the GHCDL the percentage of unique Murcko frames comes out at 69%, lower than GHCDL-V2.

### PCA-t-SNA analysis of first GHCDL, GHCDL-V2 and the small polar library

**Fig 7:**
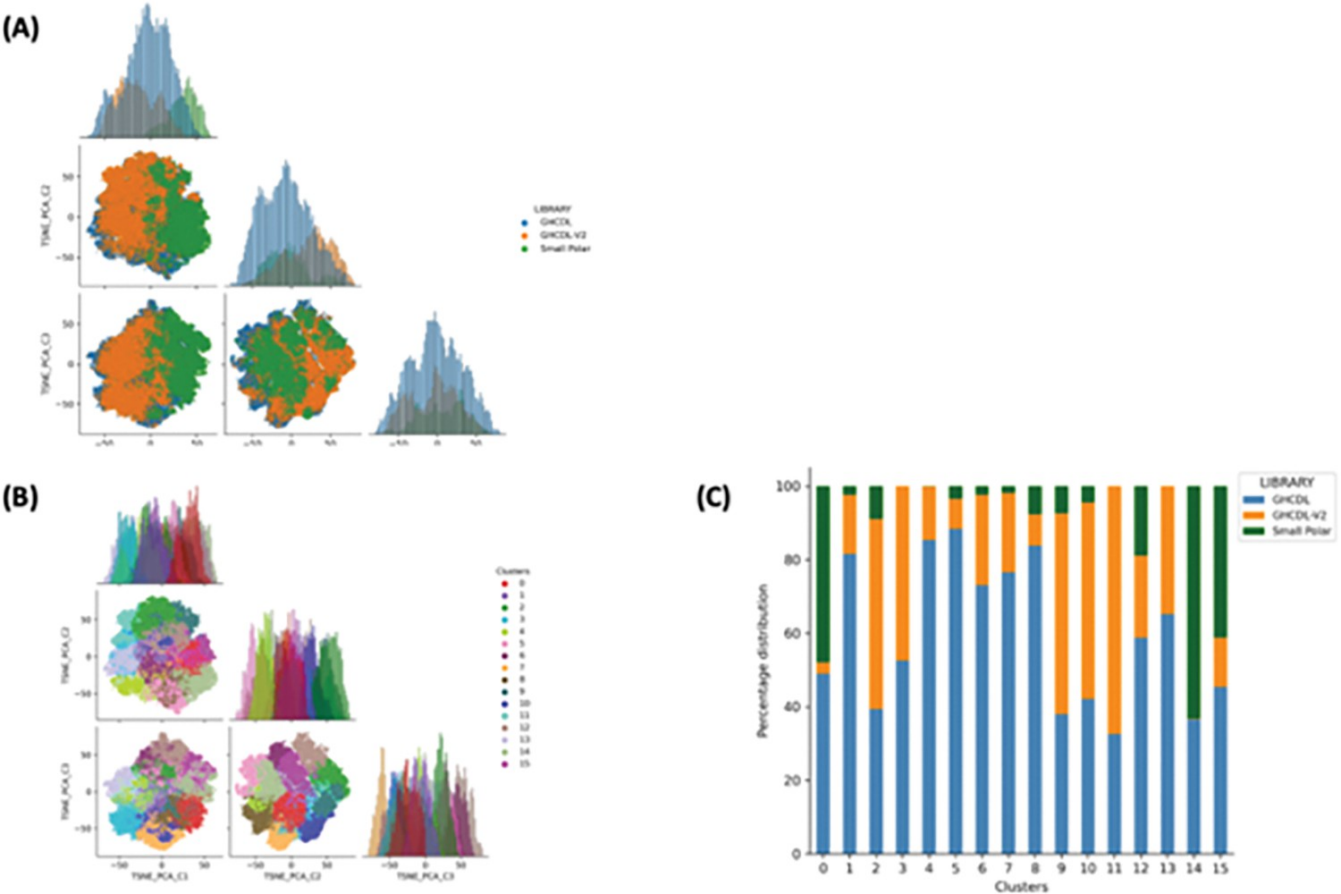
PCA-t-SNE analysis of the first GHCDL and GHCDL-V2 and the Small Polar Library, (A) Distribution of the three libraries (GHCDL, GHCDL-V2 and Small Polar) in the chemical space explored by t-SNE (PCA). Histogram shows the distribution of each dimension, (B). Kmeans clustering analysis of the three libraries and their distribution in each dimension of t-SNE(PCA) analysis, (C) contribution of each libraries to each cluster. Count expresses the number of compounds in each cluster.

**Fig 8:**
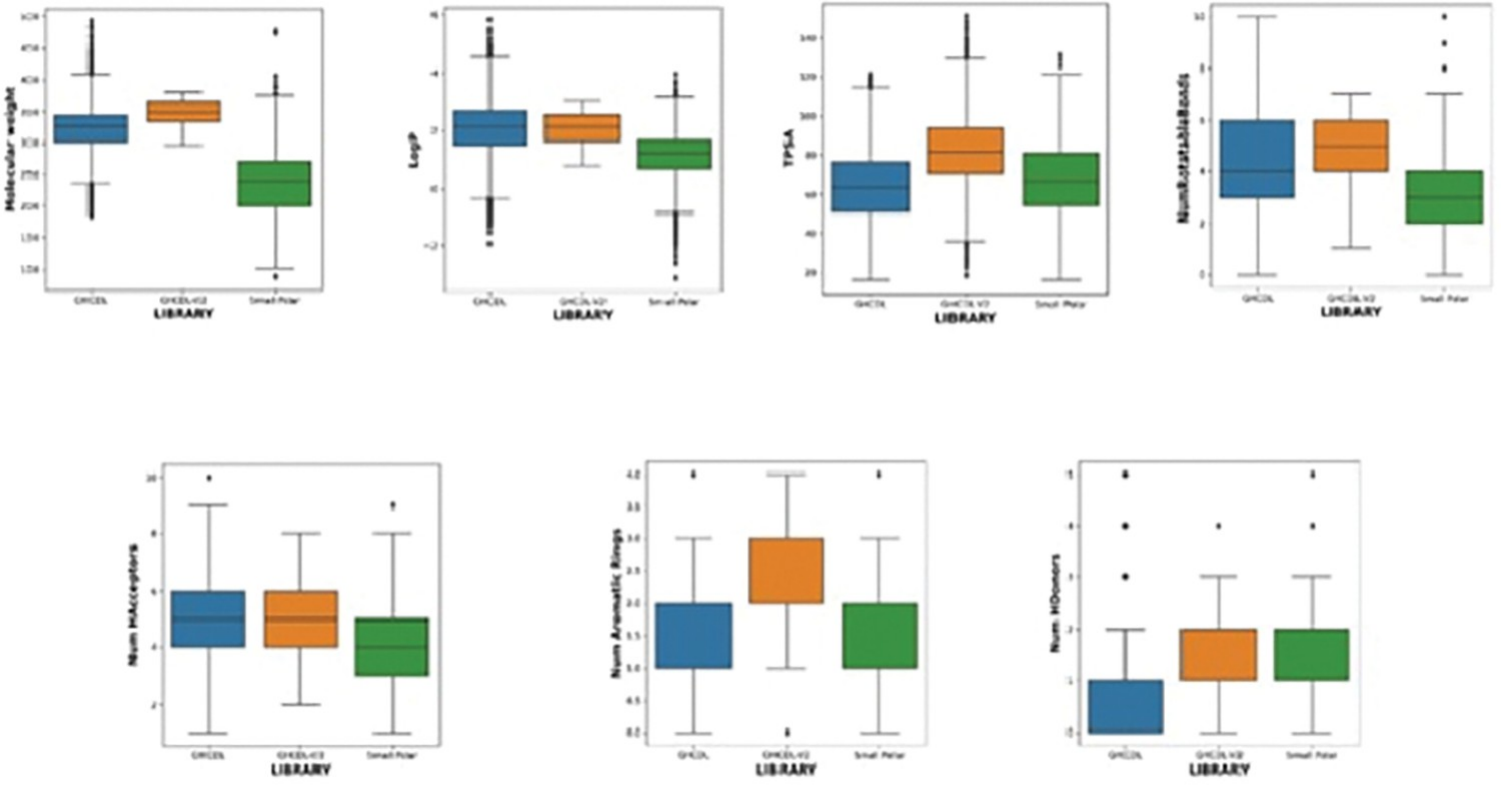
Box plots showing the different physicochemical properties of the original GHCDL, the new GHCDL-V2 and the Small Polar Library.

Smiles from the libraries were processed by RDKit software [28] and then 2D molecular descriptors (200) were calculated. A Principal Component Analysis [29] (PCA) of the first GHCDL, GHCDL-V2 and small polar libraries was carried out to reduce dimensionality to 30 and then a t-distributed Stochastic Neighbour Embedding [30] (t-SNE) analysis was applied (perplexity = 50, number of iterations = 15000) (Fig 7). Further analysis of the libraries is detailed by the box plots of the physicochemical properties shown in Fig 8. This shows that GHCDL2 is differentiated from the Small Polar Library, but the GHCDL-V2 library lies within the same space as GHCDL. Comparison of the clusters (clustered by KMeans method) shows differences between GHCDL2 and the original GHCDL. The advantages of the GHCDL-V2 over the original GHCDL are that the compounds are novel and that large numbers of analogues can readily by synthesised from within the large virtual compound space of the REAL compound collection.

### Library access

The library will be available to partner organizations for up to 25 screens in total. This will include the primary screen and then cherry picking of up to 1% of the compounds for a dose response assay. There will be a process of selection of projects for screening, owing to the number of compound sets available, to prevent duplication of screening effort and to ensure that the recipient has the ability and protocols in place in order to carry out the screening. There will be some simple rules in place for use of the library. For example, there will be no patenting allowed of compounds within the library, so that other users of the library cannot be disadvantaged. The screening data will be released into the public domain (ChEMBL), with a unique identifier, within a year of the screening being carried out. Structures of all compounds will be made available to laboratories that carry out the screen. For low throughput assays a subset of the library (~10%) will be also available.

## Conclusion

The Global Health Chemical Diversity Library v2 was selected to be a diverse set and to have chemical/physicochemical properties for a wide range of diseases and within general hit/lead-like property space. This is a set of novel compounds, currently not available from other commercial vendors. It is available for screening phenotypic and target-based screening against priority pathogens that cause major infectious diseases and also for non-hormonal contraceptives. Solids of any hits can be purchased from Enamine. For low throughput assays a subset of the library (~10%) is also available. Details of any hits will be released into the public domain to aid groups focused on drug discovery against infectious diseases of LMICs. We have decided to focus on small synthetic molecules, rather than natural products. Whist many drugs are natural products, or derivatives of natural products, there is often an issue with natural product availability and compound optimisation of natural products to address issues with potency and pharmacokinetic issues, is often very complicated. Whilst this may limit the chemical space that is covered by the library, given the vastness of chemical space, this is a pragmatic approach to facilitate the development of new hits, leads and candidates for neglected infectious diseases.

This includes the structures of the compounds in the original GHCDL and the new GHCDL2 and also additional structural alerts used in the selection of GHCDL2, in addition to those in [19].

## Supporting information

S1 Table The structures of the original GHCDL. (CSV)

S2 Table The structures of the new GHCDL. (CSV)

S3 Table New structural alerts used in the selection of the library. (XLSX)
